# *bHLH* transcription factor family identification, phylogeny, and its response to abiotic stress in *Chenopodium quinoa*

**DOI:** 10.3389/fpls.2023.1171518

**Published:** 2023-07-05

**Authors:** Guoxing Xue, Yue Fan, Chunyu Zheng, Hao Yang, Liang Feng, Xingyu Chen, Yanqi Yang, Xin Yao, Wenfeng Weng, Lingyan Kong, Chuang Liu, Jianping Cheng, Jingjun Ruan

**Affiliations:** ^1^ College of Agriculture, Guizhou University, Guiyang, Guizhou, China; ^2^ College of Food Science and Engineering, Xinjiang Institute of Technology, Aksu, China; ^3^ Agricultural Service Center of Langde Town, Kaili, Guizhou, China; ^4^ Chengdu Institute of Food Inspection, Chengdu, Sichuan, China; ^5^ The First Senior Middle School of Yuanyang County, Xinxiang, Henan, China; ^6^ Henan Institute of Technology, Xinxiang, Henan, China

**Keywords:** abiotic stress, whole genome analysis, transcription factor, *bHLH* gene family, *Chenopodium quinoa*

## Abstract

The second-largest transcription factor superfamily in plants is that of the basic helix-loop-helix (bHLH) family, which plays an important complex physiological role in plant growth, tissue development, and environmental adaptation. Systematic research on the *Chenopodium quinoa* bHLH family will enable a better understanding of this species. Herein, authors used a variety of bioinformatics methods and quantitative Real-Time Polymerase Chain Reaction (qRT-PCR) to explore the evolution and function of the 218 *CqbHLH* genes identified. A total of 218 CqbHLH transcription factor genes were identified in the whole genome, located on 18 chromosomes. A phylogenetic tree was constructed using the CqbHLH and AtbHLH proteins to determine their homology, and the members were divided into 20 subgroups and one unclustered gene. Authors also analyzed 218 *CqbHLH* genes, conservative motifs, chromosome diffusion, and gene replication. The author constructed one Neighbor-Joining (NJ) tree and a collinearity analysis map of the bHLH family in *C. quinoa* and six other plant species to study the evolutionary relationship and homology among multiple species. In addition, the expression levels of 20 *CqbHLH* members from different subgroups in various tissues, different fruit developmental stages, and six abiotic stresses were analyzed. Authors identified 218 *CqbHLH* genes and studied their biological functions, providing a basis for better understanding and further studying the *bHLH* family in quinoa.

## 1 Introduction

The bHLH transcription factor (TF) family is one of the most important in plants and plays a vital role in complex physiological processes (Murre et al., 1989). The bHLH domain comprises approximately 60 amino acids. The first part mainly comprises 13-17 amino acids, the basic N-terminal residues. The second component, the HLH domain, is mainly composed of a helix-loop-helix region of approximately 40 hydrophobic amino acids called the C-terminus, which allows bHLH proteins to form homologous or isomeric dimers (Atchley et al., 1999). According to the DNA binding and developmental characteristics of bHLH proteins, they can be divided into six groups: A, B, C, D, E, and F (Ledent and Vervoort, 2001); there are specific differences in the core domain sequences in different groups (Dang et al., 1992; Phillips, 1994; Crozatier et al., 1996; Crews, 1998; Fisher and Caudy, 1998; Ledent and Vervoort, 2001; Dang et al., 2011; Hardin, 2004).

Honkanen et al. (2018) found that the bHLH transcription factor encoded by the RSLI-like gene is necessary for regulating root cells; it is conserved in all terrestrial plants, indicating that the mechanism may be ancient and originate from a common ancestor. Pires and Dolan (2010a) found that most bHLH subgroups in angiosperms (such as Arabidopsis and rice) also exist in early differentiation populations of terrestrial plants; therefore, the diversity has been maintained for a long time, and these genes have undergone a long evolutionary process. Pires and Dolan (2010b) studied the whole genomes of some terrestrial plants and algae. They found that the diversity of bHLH proteins was established in the early terrestrial plants 440 million years ago, which is highly conserved. These characteristics may increase environmental adaptability and support morphological changes in plants.

Wang et al. (2018b) found that eight *Setaria italica bHLH* genes significantly changed expression levels under drought stress. The drought response elements have also been found in the cis-acting element analysis of these genes. Li et al. (2020) found that *CsbHLH041*, a *bHLH* transcription factor gene in *Cucumis sativus*, is an important regulator of salt and ABA (abscisic acid) tolerance. Jiang et al. (2009) also found that transcription factor AtbHLH92 in *Arabidopsis thaliana* plays a vital regulatory role in salt and osmotic stress tolerance. *AtbHLH112*, another member of the gene family, is also important in regulating plant drought resistance (Li et al., 2021). Zhao et al. (2020) found that *MdbHLH130* is an important regulator of water stress, and *CsbHLH18* was found by Geng and Liu (2018) to play a significant role in regulating cold stress. At present, bHLH transcription factor research has revealed a crucial regulatory role in response to various abiotic stresses (drought, cold, salt, etc.). According to Zhai et al. (2016), the expression level of *TabHLH39* in *Triticum aestivum* changed significantly in the roots, stems, and leaves at the seedling stage, as well as under abiotic stress (drought, salt, and cold). This indicates that bHLH transcription factors play an important role in abiotic stress resistance and plant growth.

bHLH transcription factors are closely involved in plant activities, especially in growth, fruit development, and environmental adaptation (Duek and Fankhauser, 2005; Feller et al., 2011). According to Wang et al. (2021), the overexpression of *BvbHLH93* in sugar beets can enhance antioxidant enzyme production and reduce reactive oxygen species production, significantly improving salt tolerance. *AtNHX1* and *AtNHX6* regulate salt tolerance in *Arabidopsis*, while *AtMYC2* and *AtbHLH122* regulate the expression of *AtNHX1* and *AtNHX6* under the guidance of ABA (Luo et al., 2009; Krishnamurthy et al., 2019). *MfbHLH38* from *Myrothamnus flabellifolia* was transduced into *Arabidopsis*, improving drought and salt tolerance (Qiu et al., 2020). bHLH transcription factors also directly or indirectly regulate tolerance to salt and drought in rice (Li et al., 2010; Ikeda et al., 2013; Liu et al., 2022). *PalbHLH1* and *PalMYB90* are at high expression in poplar, thereby enhancing the activity of antioxidant enzymes, releasing H_2_O_2,_ and improving its resistance to *Botrytis cinerea*. bHLH transcription factors also play an essential role in biomass synthesis; *MrbHLH1* is an indispensable synergistic gene that regulates *MrMYB1* anthocyanin synthesis. The two genes can form the *MrMYB1-MrbHLH1* complex to regulate anthocyanin synthesis (Liu et al., 2013). *DhbHLH1* and *DhMYB2* interact to regulate the production of anthocyanins in Dendrobium hybrid petals, and both *DhMYB2* and *DhbHLH1* can also induce white petals to produce anthocyanins. bHLH transcription factors also regulate lignin synthesis and have a role in hormone signal transduction, involving ABA (Li et al., 2017), jasmonic acid, brassinosteroids, salicylic acid, and ethylene (Pires and Dolan (2010a); Feller et al., 2011; Dang et al., 2011).

In 2017, the latest *C. quinoa* genome sequence was published (https://www.cbrc.kaust.edu.sa/chenopodiumdb/) (Jarvis et al., 2017), enabling us to study the evolution, development, and gene function of quinoa better. Because of the important role of bHLH transcription factors in plants, a large number of gene families have been discovered in *Arabidopsis thaliana* (Toledo-Ortiz et al., 2003), *Oryza sativa* (Li et al., 2006), *Solanum tuberosum* (Wang et al., 2018c), *Brassica rapa* (Song et al., 2014), *Solanum lycopersicum* (Sun et al., 2015), *S. italica* (Fan et al., 2021a), *Zea mays* (Zhang et al., 2018a), *T. aestivum* (Wei and Chen, 2018), *Capsicum annuum* (Zhang et al., 2020), *Fagopyrum tataricum* (Sun et al., 2020), *Sorghum bicolor* (Fan et al., 2021b), *Brachypodium distachyon* (Niu et al., 2017), and *Vitis vinifera* (Wang et al., 2018a). In this study, we identified 218 C*. quinoa* bHLH family members in the genome, divided them into 20 subgroups, and analyzed their gene structure, conserved motifs, gene distribution, phylogeny, and homology. We also examined the bHLH gene expression levels under abiotic stress and during stages of growth and development, which helps study the gene function and evolution in *C. quinoa*.

## 2 Materials and methods

### 2.1 Gene identification

The latest *C. quinoa* genome sequence was downloaded from https://www.cbrc.kaust.edu.sa/chenopodiumdb/. The bHLH family members were identified in two BLASTp searches (Altschul et al., 1997; Liu et al., 2018). First, all possible bHLH proteins were identified from all the sequences of *C. quinoa* using BLASTp (score ≥ 100, e ≤ 1e10) and the Pfam Protein Family Database (http://pfam.sanger.ac.uk/). A consistent Hidden Markov model (HMM) profile for the bHLH domain (PF00011) was obtained. We used HMMER 3.0 software (with default parameters) with a cutoff value of 0.01 (http://plants.ensembl.org/hmmer/index.html) and SMART software (http://smart.emblheidelberg.de/) to determine the existence of the bHLH domains (Bateman et al., 2000; Finn et al., 2011; Letunic and Bork, 2018), verify the results, and deduce the *C. quinoa* bHLH family members. In addition, from the ExPasy website (http://web.expasy.org/protparam/), the basic characteristics of the members of the *CqbHLH* gene family were identified: protein length, coding sequence length (CDS), isoelectric point (pI), molecular weight (MW) and subcellular localization.

### 2.2 *bHLH* gene structure

ClusterW with the default parameters was used to create multiple protein sequence alignments between 218 *CqbHLH* genes and *Arabidopsis* bHLH proteins (Thompson et al., 2003). GeneDoc software was used to manually adjust the deduced amino acid sequence of the bHLH domain. The exon/intron structure of each *CqbHLH* gene was analyzed using the gene structure display server GSDS (http://GSDS.cbi.pku.edu.cn) (Guo et al., 2007). The bHLH protein motifs were identified using the MEME server (http://meme-suite.org/tools/meme) with the following settings: 10 motifs and 6–200 residues (Bailey et al., 2009; Liu et al., 2018; Xie et al., 2018; Liu M. et al., 2019).

### 2.3 Chromosome distribution and gene replication

Using Circos and the *C. quinoa* database (https://www.cbrc.kaust.edu.sa/chenopodiumdb/) (Krzywinski et al., 2009), all *CqbHLH* genes were mapped to the 18 chromosomes and Chr00 of *C. quinoa*. The MCScan X tool kit was used to analyze gene replication events with the default parameters (Wang et al., 2012). We used TBtools’ Dual Syntony Plotter (https://github.com/CJ-Chen/TBtools) to analyze homology within six species (*A. thaliana*, *V. vinifera*, *S. lycopersicum*, *B. detachyon*, *O. sativa*, and *Z. mays*). The Ka/Ks Calculator 2. Zero was used to calculate the non-synonymous (Ka) and synonymous (Ks) values of each duplicate *bHLH* gene (Wang et al, 2010).

### 2.4 Phylogenetic analysis and classification of the *CqbHLH* gene family

All identified *CqbHLH* genes were grouped according to the classification of *bHLHTFs* in *A. thaliana*. The Jukes–Cantor model of MEGA 7.0 was used to build an NJ tree with a bootstrap value of 1000, and the BLOSUM62 cost matrix was used to allocate Genetic R11. The UniProt database (https://www.uniprot.org/) and published journals were used to obtain three dicotyledons, *A. thaliana*, *V. vinifera*, and *S. lycopersicum*, and three monocotyledons, *B. distachyon*, *O. sativa*, and *Z. mays*; protein sequences were combined with 218 CqbHLH sequences for phylogenetic analysis.

### 2.5 Bicolor plant materials, growth conditions, and abiotic stresses

Wild C. quinoa (2n = 4x = 36) is the latest sequenced *C. quinoa* variety and is widely researched. In 2021, *C. quinoa* was planted at Guizhou University. *C. quinoa* Material I was planted in a pot plant mixed with soil and vermiculite (1:1); the relative humidity in the growth chamber was 75%, sunlight lasted 16 h per day (25°C), and darkness lasted 8 h per day (20°C). When the plants grew to the seedling stage (on the 21st day), we took five samples with good growth and in similar condition, collected roots, stems, and leaves, and quickly stored them in liquid nitrogen at −80° C for further use. They were then subjected to six abiotic stress treatments, including high temperature (40°C), low temperature (4°C), flooding (of the whole plant), drought (30% PEG6000), salt (5% NaCl), and ultraviolet radiation (70 µW/cm, 220 V, 30 W). The roots, stems, and leaves samples were taken after 0, 2, and 24 h, respectively, and quickly stored in liquid nitrogen at −80°C for further use. The *C. quinoa* material II was planted in the experimental field of Guizhou University, and the cultivation and management measures were consistent with the field practice. Five quinoa plants of similar growth status and length were selected to take tissue samples of roots, stems, leaves, flowers, and fruits (middle of grain-filling stage) samples were taken at maturity and immediately placed in liquid nitrogen, and stored at −80°C. When the fruit begins to appear, fifteen plants with uniform growth were selected for mixed sampling on days 7, 14, 21, 28, and 35 when the fruits appeared. Immediately after sampling, place the sample in liquid nitrogen and store it at -80°C.

### 2.6 Total RNA extraction, cDNA reverse transcription, and qRT PCR analysis

Total RNA was extracted using a plant RNA extraction kit (TaKaRa Bio) and treated with DNase I without the RNA enzyme to remove trace DNA. qRT-PCR primers were designed using Primer Premier 5.0 software (**Table S8**) (Singh et al.,1998). The internal control was the *GAPDH* gene (Sudhakar Reddy et al., 2016). qRT-PCR was performed at least three times using SYBR Premix Ex TaqII (TaKaRa Bio). We used 2^−(ΔΔCt)^ to calculate the relative gene expression (Livak and Schmittgen, 2001).

### 2.7 Statistical and analysis

We used JMP6. Zero software (SAS Institute) to analyze all of the above data through ANOVA. The least significant difference (LSD) was compared, with a significance level of *p*<0.05. Origin 8.0 software was used to draw a histogram.

## 3 Results

### 3.1 Identification of *bHLH* genes in *C. quinoa*

We used two domain identification methods to find all the possible bHLH family members in the *C. quinoa* genome. To distinguish the genes, they were named *CqbHLH1* to *CqbHLH218* according to their chromosomal position. The characteristics of these genes, including the amino acid number, molecular weight (Mw), isoelectric point (pI), coding sequence length (CDS), domain information, and subcellular localization, are provided (**Table S1**). Among the 218 proteins, CqbHLH32 was the smallest, with only 84 amino acids, and CqbHLH86 was the largest, containing 962 amino acids; the average amino acid number was 324. The Mw of the 218 CqbHLHs ranged from 9.84 kDa (CqbHLH32) to 104.51 kDa (CqbHLH86), with an average of 35.86 kDa. The pI ranged from 4.6 (CqbHLH215) to 11.84 (CqbHLH93), with an average value of 6.78. Of these 218 CqbHLH proteins, 2 contained only the bHLH-MYC_N domain (*CqbHLH16* and *CqbHLH167*), and 192 had only the HLH domain. According to the predicted subcellular localization, 183 CqbHLHs were located in the nucleus, 13 in the chloroplast, 13 in the cytoplasm, two in the Golgi apparatus (CqbHLH62, CqbHLH70), two in the peroxisome (CqbHLH16, CqbHLH66), two in the plasma membrane (CqbHLH86, CqbHLH116), one in the mitochondria (CqbHLH177), one in the endoplasmic reticulum (CqbHLH151), and one in the extracellular matrix (CqbHLH40). The proportion of *CqbHLH* genes in *C. quinoa* to the total gene number was approximately 0.49%, similar to that in *O. sativa* (0.44%) and *S. lycopersicum* (0.46%) and less than that in *A. thaliana* (0.59%) (Toledo-Ortiz et al., 2003; Li et al., 2006; Sun et al., 2015).

### 3.2 Multiple sequence alignment, phylogenetic analysis, and classification of CqbHLH proteins

**Figure 1:**
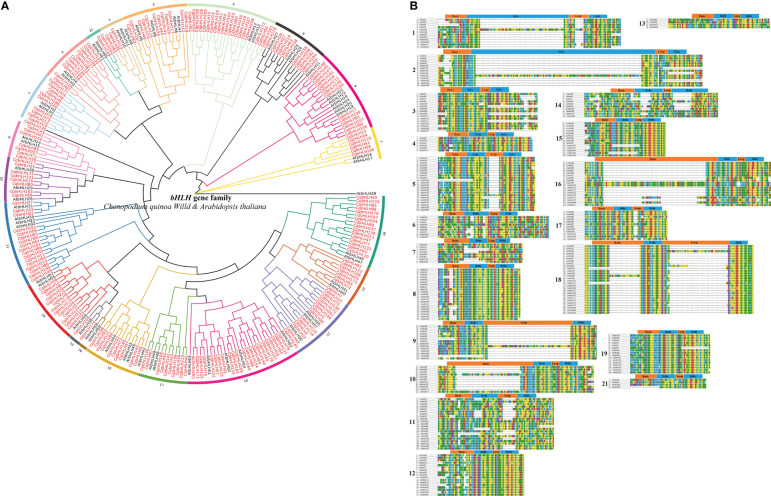
**(A)** A rootless phylogenetic tree showing the relationship between the bHLH domains of *C. quinoa* and *A. thaliana*, where AtbHLH represents a bHLH protein of *A. thaliana*. **(B)** Multiple sequence alignment between *C*. *quinoa* and 20 subgroup of *A. thaliana*. *C. quinoa* lacks the subgroups 20. The boundary and location of the basic helix loop helix are described at the top of the subgroup.

Using the 218 identified CqbHLH proteins and other 61 Arabidopsis bHLH proteins, we constructed a phylogenetic tree with a bootstrap value of 1000 through Neighbor-Joining (NJ) (**Figure 1A**; **Tables S1**, **S2**). Based on the topological structure of the tree and the classification method proposed by Pires and Toledo-Ortiz (Pires and Dolan (2010a); Toledo-Ortiz et al., 2003), we divided the 218 CqbHLH proteins into 20 subgroups and one ungrouped protein (CqbHLH135), consistent with the classification group of bHLH proteins in *Arabidopsis*, indicating that they were retained during long-term evolution. However, subgroup 20 was absent from the bHLH family of *C. quinoa*, indicating that it may have been lost or undifferentiated in the long-term evolution of *C. quinoa*. Among the 20 subgroups of *C. quinoa*, subgroup 18 had the most members (23 CqbHLHs), while subgroups 13 and 21 had the least members (2 CqbHLHs). The phylogenetic tree showed that some CqbHLHs were closely bound to AtbHLHs (bootstrap support ≥ 80). The collinearity analysis of *C. quinoa* and *A. thaliana* revealed the homology of CqbHLHs with AtbHLHs, speculated to have similar functions.

The bHLH domains of the selected *A. thaliana* and *C. quinoa* family members were sequenced (**Figure 1B**); most of the bHLH domains in *C. quinoa* span approximately 53 amino acids. There were 17 amino acids in the basic region, 15 in the helix region, and six in the loop region (Toledo-Ortiz et al., 2003). Meanwhile, some *C. quinoa* bHLH family members’ conservative domains also showed a significant amino acid number increase (such as the helix region of CqbHLH16 and CqbHLH129). The loop is the most divergent region of this domain, especially in subgroups 1, 5, 9, 11, 17, and 18. The same is true for bHLH proteins in other plants, including *A. thaliana* (Toledo-Ortiz et al., 2003), *S. lycopersicum* (Sun et al., 2015), *S. italic* (Fan et al., 2021a) and *F. tataricum* (Sun et al., 2020).

### 3.3 Analysis of conserved domains, gene structure, and cis-acting elements of *CqbHLH* genes

**Figure 2:**
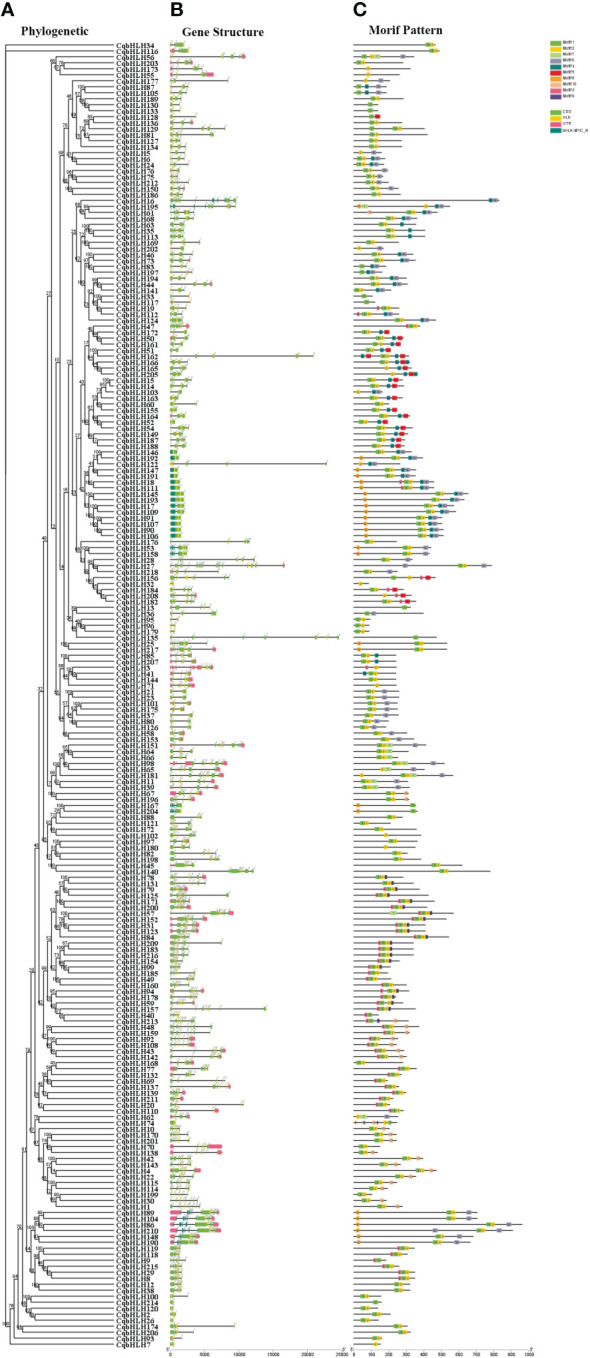
Phylogenetic tree, gene structure, and motif distribution of 218 *bHLH* genes in *C. quinoa*. **(A)** The phylogenetic tree is constructed using the NJ (Neighbor- Joining) method, with 1000 replicates on each node. **(B)** Pink area: UTR (untranslated area), yellow area: CDS (coding sequence), and yellow/dark green area: bHLH domain (HLH, bHLH-MYC_N). **(C)** The colored area represents the conservative motifs (motifs 1–10) of 218 CqbHLHs, and the black line represents the relative protein length.

Understanding the intron-exon structures of *CqbHLH* genes will help explore their physiological functions. A comparison of the number and location of exons and introns shows that the 218 *CqbHLHs* genes have between one and 19 exons (**Figures 2A, B**; **Tables S1**, **S8**); 14 genes (6.42%) contain one exon, and the rest have two or more exons. The 14 intron-free genes belong to three subgroups (8, 14, and 19) but are mainly found in subgroup 8 and 19, among the 42 genes with introns, four are the most common. *CqbHLH27* has the most introns (18), while members of Group 8 had no introns or only one intron. Group 9 had the most variation in exon number: one intron in *CqbHLH32*) and 18 introns in *CqbHLH27*. Through comparative analysis, we found that subgroup 11 showed the greatest diversity in intron numbers.

To further study the characteristic regions of the 218 CqbHLH proteins, we used the online tool MEME (https://meme-suiteorg/meme/tools/meme). The conservative motifs of the 218 CqbHLH proteins were analyzed, and ten were selected. **Figure 2C** shows that motifs 1 and 2 were widely distributed and the most numerous in CqbHLHs, whereas motif 7 was the least distributed in the CqbHLHs. CqbHLH members in the same subgroup usually have similar motif compositions. For example, the members in subgroup 5 mainly include motifs 1, 2, 4, and 5. Subgroup 8 mainly includes motifs 1, 2, 6, and 9. Some motifs mostly existed in specific subgroups, such as motif 9 in subgroup 18. Motifs 1 and 2 were found in most of the subgroups. After further sequence analysis, we found that the motifs have specific arrangement characteristics and positional relationships; for example, motif 6 is only found at the end of subgroups 1, 8, 9, 10, 11, 12, and 21; in the subgroup 18, there is a specific arrangement of motifs 3, 1, 2 and 9. Studying the conserved motifs of proteins will help us to research their functions systematically. The functions of most of these CqbHLH proteins are yet to be proven, but their phylogenetic results support the groupings of the *C. quinoa bHLH* gene subgroups.

**Figure 3:**
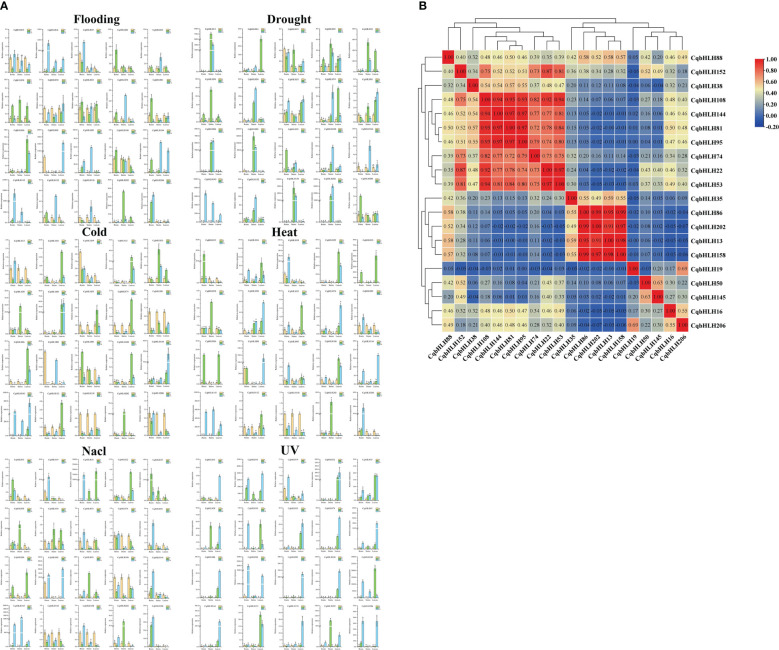
**(A)** The gene expression patterns of 20 *CqbHLH* genes in the roots, stems, and leaves of young *C*. *quinoa* seedlings treated with 0 h, 2 h, and 24 h abiotic stresses (UV, flooding, drought, NaCl, heat, and cold) were detected by qRT-PCR. The error value is obtained from the three measurements. The lowercase letters above the bar graph represent significant differences between different letters (p < 0.05, LSD). **(B)** Number > 0: positive correlation; Number < 0: negative correlation; 0.05 represents a significant correlation.

We analyzed the cis-acting elements of 218 *CqbHLH* gene initiation codons, 2000 bp upstream (**Table S3**). Analysis of the cis-acting elements of the 218 *CqbHLHs* genes showed that light- and hormone-related elements were the most abundant; ABA response elements were the most abundant among the hormone-related elements. The hormone-related elements include gibberellin, salicylic acid, auxin, and methyl jasmonate response elements. Concerning environmental pressures, the *CqbHLH145* promoter contained low-temperature response elements, and *CqbHLH145* also showed high expression levels under cold stress (**Figure 3A**), indicating direct or indirect regulation of the physiological metabolism of quinoa at low temperatures.

### 3.4 *CqbHLH* chromosome distribution and gene replication

**Figure 4:**
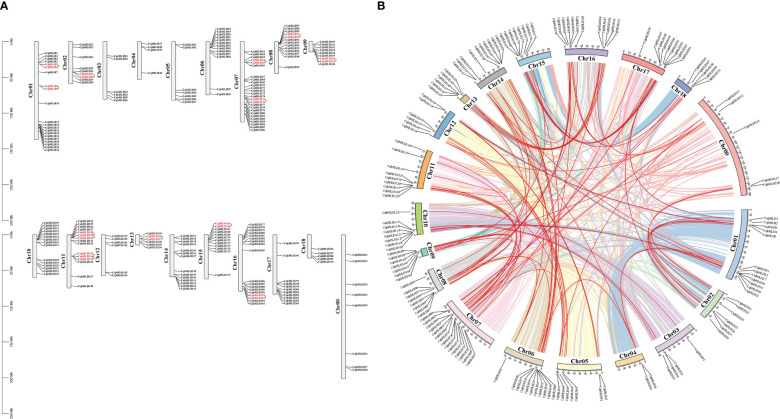
**(A)** Location map of 218 *bHLH* genes on *C. quinoa* chromosomes. The scale on the left side of the chromosome indicates its length. To the left side of the chromosome is its number; to the right side is the *C*. *quinoa bHLH* gene contained in the chromosome; the red *CqbHLH* is a tandem repeat gene. **(B)** The schematic diagram of the distribution of *C. quinoa* genes on the chromosomes and the relationship between chromosomes. Different color lines inside represent gene pairs between different chromosomes; red lines represent *C. quinoa bHLH* gene pairs; the *C. quinoa bHLH* genes are indicated outside the chromosome, inside of which is the chromosome number.

A physical location map of *CqbHLH* was created (**Figure 4A**; **Table S4**). The 218 *CqbHLH* genes were unevenly distributed on the 18 chromosomes (Chrs) and Chr00. Each gene is named according to its physical location from top to bottom on Chr1 to Chr18 (Chr00 comprises the segments that could not be located on chromosomes). Among these, Chr07 had the largest number (26 genes, ~11.93%), followed by Chr01 (21 genes, ~9.63%), and Chr04 had the lowest content (4 genes, ~1.83%). Chr16 contained 18 (~8.26%) *CqbHLH* genes; Chr11 and Chr10 contained 15 (~6.88%) *CqbHLH* genes; Chr14 contained 14 (~6.42%) *CqbHLH* genes; Chr08 and Chr06 contained 13 (~5.96%) *CqbHLH* genes; Chr15 contained 12 (~5.50%) *CqbHLH* genes; Chr15 contained 11 (~5.05%) *CqbHLH* genes; Chr09 and Chr02 contained 9 (~4.13%) *CqbHLH* genes; Chr00 contained 8 (~3.67%) *CqbHLH* genes; Chr05 contained 7 (~3.21%) *CqbHLH* genes; Chr03, Chr12, and Chr13 each contained 6 (~2.75%) *CqbHLH* genes; Chr18 contains five (~2.29%) *CqbHLH* genes. Most of the *CqbHLH* genes were distributed on the 18 chromosomes and at the end of Chr00. We observed many *CqbHLH* gene replication events; chromosomal regions within 200 kb of two or more identical genome regions were defined as tandem duplication events (Chen et al., 2017). Twelve tandem repeats were found on Chr01, Chr02, Chr07, Chr08, Chr09, Chr11, Chr13, Chr15 and Chr16, involving 26 *CqbHLH* genes. Among them, *CqbHLH128*, *CqbHLH129*, *CqbHLH130*, *CqbHLH133*, *CqbHLH134*, and *CqbHLH135* on Chr11 had two tandem repeat events (*CqbHLH129* and *CqbHLH128*/*CqbHLH130*; *CqbHLH134* and *CqbHLH133*/*CqbHLH135*). All genes with tandem repeats belonged to the same subgroup (except for *CqbHLH135*). For example, *CqbHLH5* and *CqbHLH6* are tandem repeats clustered in subgroup 1.

In addition, the gene fragment repeat analysis showed that there were 152 similar sites in *CqbHLH*, and 95 pairs of fragments were duplicated. A total of 152 (69.72%) homologous genes were identified in the *CqbHLH* gene family, indicating an evolutionary relationship between *bHLH* members (**Figure 4B**; **Table S5**). The *CqbHLH* gene was unevenly distributed among the 19 *C. quinoa* linkage groups (LGs). Some LGs (LG07) had more *CqbHLH* genes than others (LG04 and LG03). The *CqbHLH* gene of LG07 is the most (15), whereas that of LG04 is the least (2). Further analysis of the genes showed that fragments of repeated *CqbHLHs* were connected within their subgroups, except for *CqbHLH13*/*CqbHLH22*, *CqbHLH7*/*CqbHLH143*, *CqbHLH32*/*CqbHLH122*, *CqbHLH42*/*CqbHLH208*, and *CqbHLH184*/*CqbHLH47*. These results indicate that some *CqbHLH* genes may result from replication events, which play an essential role in the evolution of *C. quinoa* and amplify the *CqbHLH* gene family.

### 3.5 Collinearity analysis of CqbHLH

**Figure 5:**
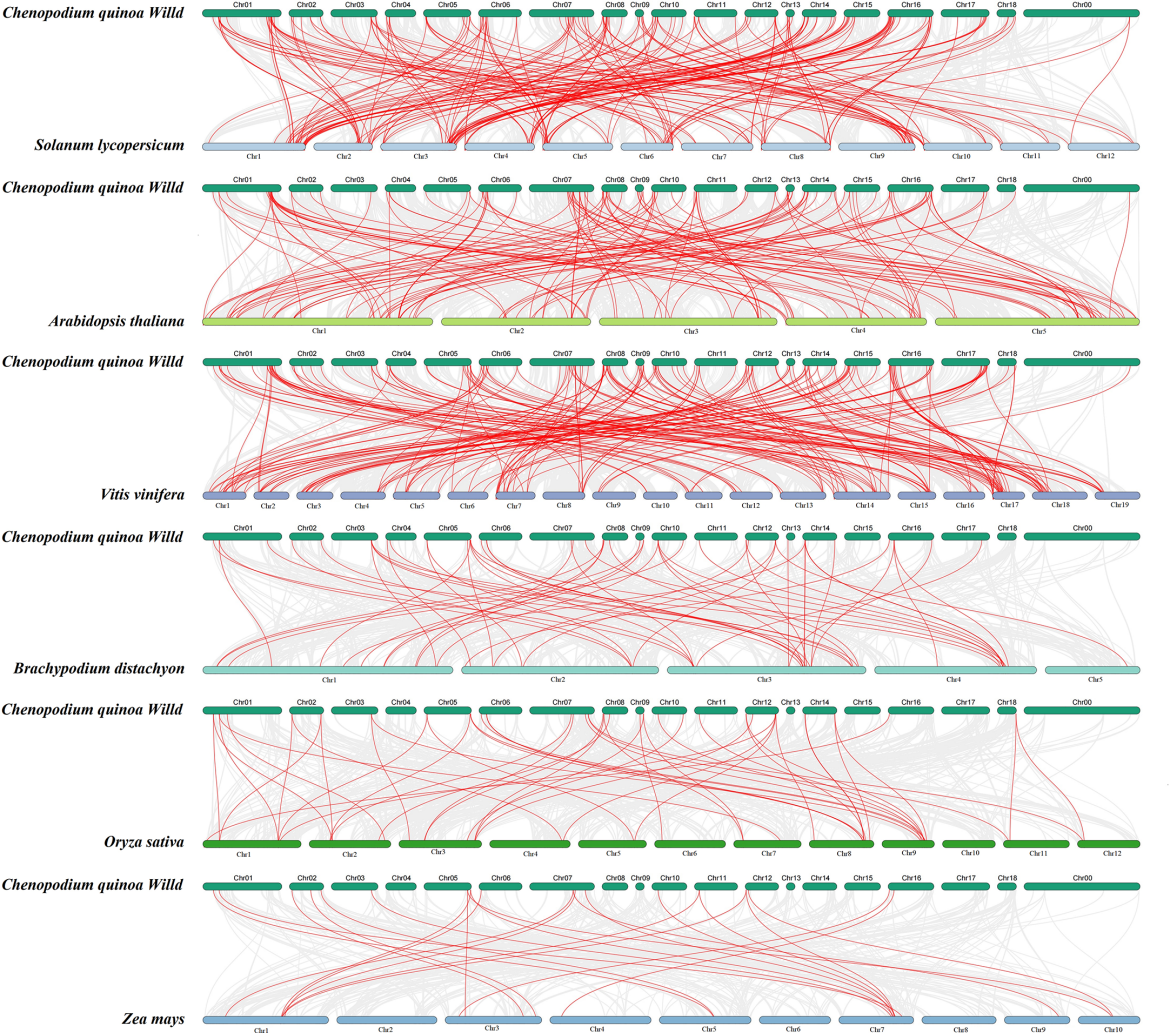
The collinearity diagram of *bHLH* genes in quinoa and six other representative plants: three dicotyledons (tomato, Arabidopsis, and grape) and three monocotyledons (two spike grass, rice, and corn). The gray line represents the collinear gene pairs between the two species, and the red line represents the *bHLH* gene pair.

To further determine the phylogenetic mechanisms of the bHLH family, we constructed a collinearity diagram of *C. quinoa* and six representative species (three representative dicotyledon plants: *A. thaliana*, *S. lycopersicum*, and *V. vinifera*, and three representative monocotyledon plants: *B. distachyon*, *O. sativa*, and *Z. mays*) (**Figure 5**; **Table S6**). A total of 147 *CqbHLH* genes were found to have a collinear relationship with the genes of *A. thaliana* (82), *V. vinifera* (90), *S. lycopersicum* (96), *B. distachyon* (33), *O. sativa* (28) and *Z. mays* (15). At the same time, the homologous logarithms for the six species are *A. thaliana* (135), *V. vinifera* (184), *S. lycopersicum* (191), *B. detachyon* (57), *O. sativa* (48) and *Z. mays* (23). Some *CqbHLH* genes are associated with at least four synthetic gene pairs (particularly *C. quinoa* and *B. detachyon*), such as *CqbHLH33* and *CqbHLH117*. *CqbHLH* and *A. thaliana* (52.0%), *B. detachyon* (52.63%), *O. sativa* (56.25%), and *S. lycopersicum* (59.68%) contained two or more synthetic gene pairs, accounting for more than 50% of the total homologous logarithm, suggesting that these genes are essential in evolution.

As expected, some *CqbHLH* genes only had homologous gene pairs with three dicotyledons, such as *CqbHLH55*, *CqbHLH58*, *CqbHLH71*, and *CqbHLH72*. They all have at least one pair of homologous genes with three selected representative dicotyledons, indicating that these genes may have gradually formed after the independent differentiation of dicotyledons. We also found that some of the *CqbHLH* genes pair with at least one homologous gene within the six species, such as *CqbHLH139*, *CqbHLH30*, *CqbHLH22*, *CqbHLH74*, *CqbHLH117*, *CqbHLH47*, *CqbHLH1*, and *CqbHLH33*, indicating that they may be essential primitive genes that have not been lost or shown strong differentiation during the long-term evolution of *C. quinoa*.

### 3.6 Evolutionary analysis of bHLH Proteins in *C. quinoa* and six other species

**Figure 6:**
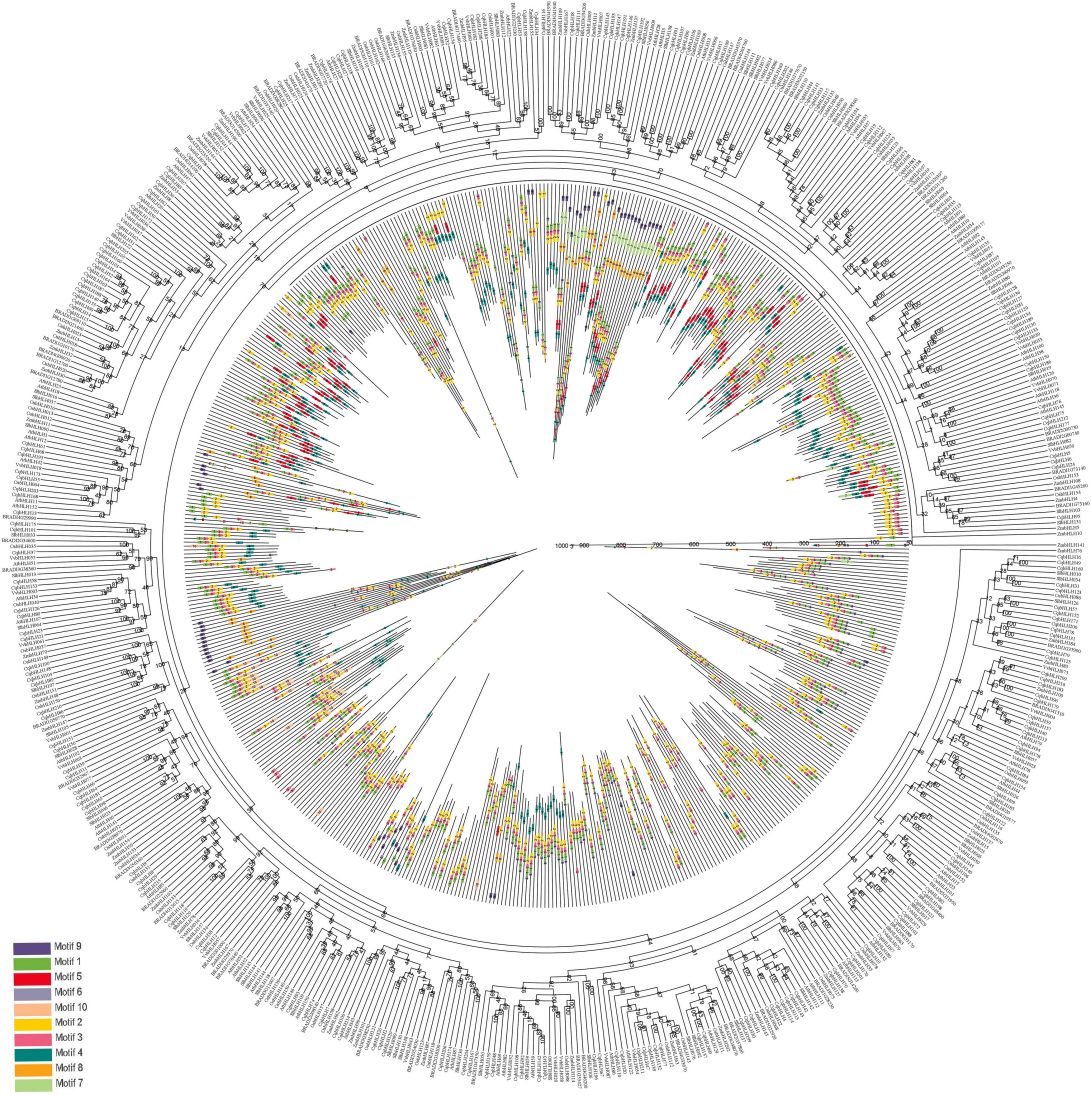
Phylogenetic tree and conserved motifs (1–10) bHLH proteins in quinoa and six representative plants (three dicotyledons representing *S. lycopersicum*, *A. thaliana*, and *V. vinifera*; three monocotyledons representing *B*. *distachyon*, *O. sativa*, and *Z. mays*). The outer circle is the quinoa and six species bHLH rootless phylogenetic tree, and the inner circle is the distribution of conservative motifs in and the relative length of bHLH proteins.

To analyze the evolutionary relationship between the *C. quinoa* protein triple helix family and six other plants (*A. thaliana*, *B. distachyon*, *S. lycopersicum*, *V. vinifera*, *O. sativa*, and *Z. mays*), we used the MEME web server and the Genes R11 method to construct a rootless NJ tree with ten conserved motifs from the amino acid sequences of the 218 CqbHLH proteins and the bHLH proteins (355) from the other six plants (**Figure 6**; **Tables S7**, **S8**). The distribution of *CqbHLHs* in phylogenetic trees is relatively scattered, while the CqbHLH protein is more inclined to *S. lycopersicum*. The bHLH protein aggregation of *S. lycopersicum* is shown in **Figure 3**. The proteins of the seven plants mainly contained motifs 1, 2, and 3. Motifs 1, 2, and 3 were almost all arranged in the same way in the seven species. Subgroup 8 contained the most: Motifs 9, 7, 8, 1, 3, 2, 5, and 4. bHLH proteins from the same branch of rootless NJ trees usually had similar motifs and motif arrangements; compared with the other four selected representative plants, bHLH proteins tended to be on the same branch in *B. detachyon* and *S. lycopersicum*, showing that their relationship with CqbHLH proteins may be close, compared with the other four plants.

### 3.7 CqbHLH expression patterns in different plant organs and at different times in fruit stage

**Figure 7:**
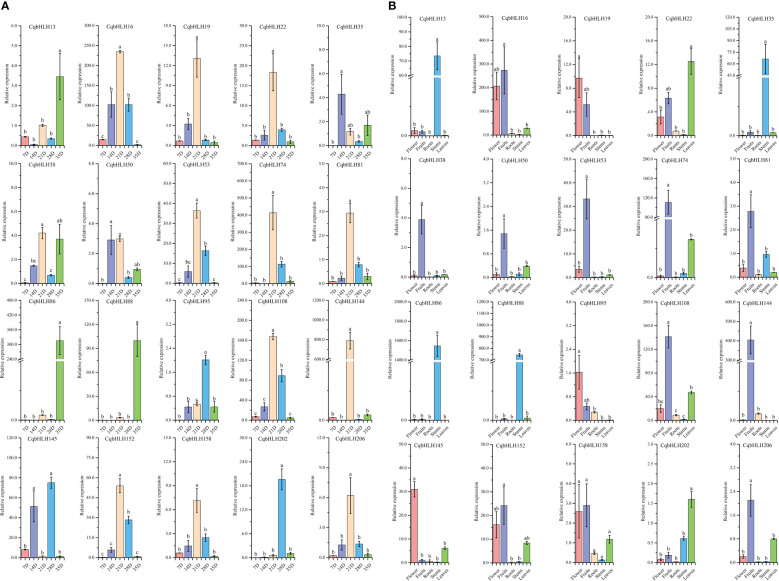
**(A)** The expression patterns of 20 CqbHLH genes at different stages after fruits appeared were detected by qRT-PCR. Among them, 7 D, 14 D, 21 D, 28 D, and 35 D represent the 7th, 14th, 21st, 28th, and 35th days after fruits appeared. **(B)** The tissue-specific expression patterns of 20 *bHLH* genes in roots, stems, leaves, flowers, and fruits were detected by qRT-PCR. The error value is obtained from the three measurements. The lowercase letters above the bar graph represent significant differences between different letters (p< 0.05, LSD).

To study the role of *CqbHLHs* in the growth period of *C. quinoa*, we selected 20 representative genes from the CqbHLH gene family according to the homologous relationship between *CqbHLHs* and *AtbHLH* genes and the evolutionary relationship of NJ tree. We used qRT-PCR (quantitative real-time polymerase chain reaction) technology to detect the gene relative expression of these members in different organs during the appropriate development stages of *C. quinoa* plants (**Figures 7A, B**). The results showed different expression patterns in the selected 20 *CqbHLH* genes. At different times of fruit development (**Figure 7A**), it was found that among the 20 *CqbHLH* genes, most of them (*CqbHLH16*, *CqbHLH19*, *CqbHLH22*, *CqbHLH38*, *CqbHLH50*, *CqbHLH53*, *CqbHLH74*, *CqbHLH81*, *CqbHLH108*, *CqbHLH144*, *CqbHLH152*, *CqbHLH158* and *CqbHLH206*) showed the highest expression in 21 D (day). Three genes (*CqbHLH13*, *CqbHLH86*, and *CqbHLH88*) were found to have the highest expression in 35 D. No genes were found to have the highest expression at 7 D. In the different organs (**Figure 7B**), it was found that most of the 20 *CqbHLH* genes (*CqbHLH16*, *CqbHLH38*, *CqbHLH50*, *CqbHLH53*, *CqbHLH74*, *CqbHLH81*, *CqbHLH108*, *CqbHLH144*, *CqbHLH152*, *CqbHLH158* and *CqbHLH206*) were highest expressed in fruits. In the stem, four genes (*CqbHLH13*, *CqbHLH35*, *CqbHLH86*, and *CqbHLH88*) showed the highest expression. Some genes have been found to exhibit typical tissue expression specificity, which may be related to the physiological pathways in which they participate.

### 3.8 Effects of different treatments on *CqbHLH* expression

To study the effects of abiotic stresses under different stresses on the *CqbHLH* gene, we carried out six abiotic stress treatments: strong ultraviolet radiation stress (UV), flooding stress (flooding), drought stress (drought), salt stress (NaCl), heat stress (heat) and cold stress (cold), and detected the gene expression of 20 *CqbHLH* genes selected under the six stresses by qRT-PCR (**Figure 3A**). We found that different stresses induced or inhibited the expression of some genes differently and that significant changes occurred in the early stage of stress. For example, *CqbHLH145* is a high expression under six types of stress. In the roots, stems and leaves, its expression was significantly upregulated under cold stress; however, under intense UV radiation, it was significantly upregulated in roots and stems. In contrast, it was significantly upregulated and then significantly downregulated in leaves. Under drought stress, *CqbHLH158* was significantly upregulated in the stems, whereas under cold stress, *CqbHLH158* was downregulated in the roots, stems, and leaves. Under flooding stress, *CqbHLH16* was upregulated in the roots, stems, and leaves, whereas *CqbHLH108* was downregulated in the roots, stems, and leaves. Interestingly, we found that the *CqbHLH202* gene was upregulated and then downregulated in the stems under cold and heat stress, possibly indicating that the *CqbHLH202* gene is crucial for temperature regulation. *CqbHLH13* had the opposite expression pattern: it was downregulated in cold-stressed roots and then upregulated back to normal levels. Under heat stress, however, the expression levels first increased and then decreased to normal. We studied the correlation between the expressions of the 20 *CqbHLHs* genes (**Figure 3B**). There were two obvious positive correlation regions, and each contained four genes. Area I includes *CqbHLH108*, *CqbHLH144*, *CqbHLH81* and *CqbHLH95*. Area II included *CqbHLH86*, *CqbHLH202*, *CqbHLH13* and *CqbHLH158*. However, there was also a negative correlation; for example, between *CqbHLH16* and *CqbHLH206* and *CqbHLH86*, *CqbHLH202*, *CqbHLH13*, and *CqbHLH158*.

## 4 Discussion

Based on the whole genome, studying the TF family of a species is conducive to the systematic exploration of the genes and their functions in the long-term development of this species. We identified 218 C*. quinoa bHLH* genes, whose proteins showed remarkable differences in amino acid number; the longest (CqbHLH86) was 962 amino acids, and the shortest (CqbHLH32) was 84 amino acids, indicating significant variability (Atchley et al., 1999; de Martin et al., 2021). The *C. quinoa* bHLH subgroup variation is mainly in the basic region, indicating its importance in the bHLH domain (Atchley et al., 1999; Toledo-Ortiz et al., 2003). The basic region determines the determining activity of the *bHLH* gene and forms homodimers or heterodimers with other transcription factors (Heim et al., 2003; Toledo-Ortiz et al., 2003). The basic region can be determined by whether the *CqbHLH* gene has DNA-binding activity and can form homodimers and heterodimers (Heim et al., 2003; Toledo-Ortiz et al., 2003). Genes with DNA activity can be further divided into E-box and Non-E-box genes, and E-box can be subdivided into G-box and Non-G-box, according to the essential amino acid residues in the basic region (Atchley et al., 1999; Toledo-Ortiz et al., 2003). In the *C. quinoa* bHLH family, there were 11 (5.04%) CqbHLHs without DNA-binding activity; 32 (14.68%) CqbHLHs were classified as non-E-box proteins and 175 (80.28%) CqbHLHs were classified as E-box proteins. Among the 175 E-box proteins, 174 (79.82%) were classified as G-box proteins, and one (0.46%) CqbHLH was classified as a non-G-box protein. Among the 20 CqbHLH subgroups, we found that subgroup 8 was the most conservative, while subgroup 11 was the most variable. This situation is the same as that of the subgroup 8 and 11 of the bHLH family species of the dicotyledon *A. thaliana* (Toledo-Ortiz et al., 2003) and monocotyledon *O. sativa* (Carretero-Paulet et al., 2010). Monocotyledonous *S. italica* and *S. bicolor* had conservative the subgroup 8 and 11 (Fan et al., 2021a; Fan et al., 2021b). In contrast, the monocotyledonous *B. distachyon* had the most conservation in subgroup 8 (Niu et al., 2017); subgroup 11 showed greater variation, indicating no specificity between dicotyledons and monocotyledons and suggesting that the subgroup 11 of CqbHLHs may have greater functional differences.

We constructed a phylogenetic tree using 218 CqbHLHs and AtbHLHs. Except for subgroup 20, we found at least one CqbHLH subgroup for each AtbHLH subgroups. This indicates that the bHLH family differentiated before *Arabidopsis* and *C. quinoa* diverged. Although the number of members and subgroups of the bHLH family in different species is quite different, they may play a similar basic role in development, adaptation, and evolution. For example, in *Hordeum vulgare* (Ke et al., 2020a), *B. napus* (Ke et al., 2020b), *Raphanus sativus* (Wang et al., 2022), and *Nicotiana tabacum* (Bai et al., 2020), it was found that the *bHLH* genes in these species are significantly expressed under abiotic stress, proving that the bHLH transcription factors play an essential role in plant development, adaptation, and evolution. In the NJ tree constructed using *A. thaliana* data (**Figure 1**), we divided *C. quinoa* into subgroup 1–21, but no subgroup 20 was found. However, in *A. thaliana* (Toledo-Ortiz et al., 2003), *S. lycopersicum* (Sun et al., 2015), and *B. napus* (Ke et al., 2020b), subgroup 20 was not lost. Therefore, based on the gene structure and evolutionary analysis of *A. thaliana*, *O. sativa*, and *C. quinoa* bHLH itself, we speculate that during the differentiation process, a certain subgroup replaced subgroup 20 and exercised its biological functions, or that subgroup 20 did not differentiate or get lost due to environmental selection. In addition, one *CqbHLH* had no obvious cluster, which indicates that *C. quinoa* may have a new evolutionary direction. In terms of gene structure, we found that *CqbHLH135* was similar but different from subgroup 16. However, *CqbHLH135*, *CqbHLH133*, and *CqbHLH134* were tandem repeats. Therefore, we speculate that *CqbHLH135* may have differentiated from subgroup 2 earlier. It is similar to subgroup 16 in gene structure but has its unique function during differentiation. *S. italica* has its subgroup, but the function of these special members in forming their subgroups has not been clarified (Fan et al., 2021a).

In the gene structure analysis, we found that some members had fewer introns than others of the same subgroup; they may have been lost during long-term evolution or reduced in number due to environmental selection, such as *CqbHLH7* and *CqbHLH74* of subgroup 14. In plants, the number of introns is related to the expression level, and a more compact gene structure may be conducive to rapid gene expression upon stimulation (Wray et al., 2003). The expansion of this gene family occurs mainly through gene replication (Schwechheimer et al., 1998; Li et al., 2006; Wang et al., 2018c). The *C. quinoa* bHLH family contains 12 tandem repeats, including 26 *CqbHLH* genes and 95 pairs of fragment repeats, suggesting that the role of fragment repeat may be more significant than that of tandem duplication.

The bHLH TFs have many members, second only to the MYB TFs, which have essential and extensive functions (Buck and Atchley, 2003; Hao et al., 2021). However, research mainly focuses on two model plants, Arabidopsis and rice, and the bHLH family of other plants has not yet been systematically studied. We found that subgroup 18 has the largest number of members (10.55%), similar to the bHLH family of *Arabidopsis* (Toledo-Ortiz et al., 2003). The *CqbHLH145* gene of the selected subgroup 18 members showed a significant increase in gene expression during growth, development, and biological stress and accounted for a large proportion (13.68%) of the fragment repeat in the *C. quinoa* bHLH family. Therefore, we speculate that, compared with other species, the subgroup 18 genes may play a critical role in the growth and development of *C. quinoa*, but this has not been proven. In addition, we selected 20 *CqbHLH* genes from the 20 subgroups (there were no *C. quinoa* bHLH members in subgroup 20) to study their responses to six abiotic stresses and reproductive stages. Although there were significant differences in the expression levels between the genes, some showed two or more significant differential expression levels. For example, *CqbHLH202* of subgroup 21 is high expression under NaCl stress. In *A. thaliana*, *AtbHLH92*, also in subgroup 21, is a high expression under NaCl stress (Jiang et al., 2009). In *C. quinoa* leaves under cold stress, 11 genes were significantly upregulated, and eight were significantly downregulated, indicating that *CqbHLH* genes have synergistic or antagonistic effects under various stresses. Fan et al. (2021a) found that in *S. italica*, there was also up/downregulated expression of various *SibHLH* genes under abiotic stress. We found that the *CqbHLH145* gene was significantly upregulated under six stresses, which was not found in *A. thaliana* and is worthy of further study (Hao et al., 2021).

Cis-acting elements regulate the activity of target genes by binding to trans-acting factors, thereby regulating gene expression (Liu et al., 2019). In this study, 218 cis-acting elements in the promoter region of the *CqbHLH* genes were predicted, and it was found that the most hormone responsive elements in the promoter region of the *CqbHLH* genes were the abscisic acid (ABA) responsive elements. *bHLH* gene is usually involved in ABA signaling pathways to regulate plant drought resistance, such as *bHLH122* in *A. thaliana*, which enhanced drought resistance in *A. thaliana* by inhibiting the catabolism and metabolism of ABA (Liu et al., 2014). In *C. quinoa*, it has been found that under drought stress, the *bHLH* gene is highly expressed in both the stems and leaves of *C. quinoa*. This may be due to the involvement of the *bHLH* gene in the ABA signaling pathway, increasing ABA content, or reducing ABA degradation, to improve the drought resistance of *C. quinoa*. Other studies have found that *bHLH* is also related to the development of leaf stomata (Yang et al., 2017; Zhang et al., 2018b), while more *bHLH* genes are highly expressed in *C. quinoa* leaves. Therefore, it is speculated that the *bHLH* gene of *C. quinoa* is also involved in the development of leaf stomata, or in regulating the opening and closing of leaf stomata to improve drought resistance of *C. quinoa*. This is also consistent with the physiological characteristics of *C. quinoa*, a plant with strong drought resistance.

We found that 20 *CqbHLH* genes were positively regulated in the gene expression heat map, indicating that multiple *CqbHLH* genes may be co-expressed to perform this physiological function. In addition, it is crucial to study the role of the bHLH family in the reproductive organs of *C. quinoa*. We investigated the expression of 20 *bHLH* genes at different times in the main organs of *C. quinoa* at the reproductive stage. The *CqbHLH* genes also play important roles in the reproductive development of *C. quinoa*. For example, the expression level of *CqbHLH144* in the fruits was significantly higher than in the roots, stems, leaves, and flowers. Gene expression reached its highest level on the 21st day after flowering. *CqbHLH145*, like *CqbHLH144*, exhibits significant changes in gene expression during reproductive development. These two genes are likely to regulate the reproductive developmental stage of *C. quinoa*. In the maize *bHLH* family, the yeast two-hybrid test showed that *ZmbHLH23* and *ZmbHLH180* have a synergistic expression (Zhang et al., 2018a). Moreover, according to the gene correlation heat map, we found that two genes (*CqbHLH145* and *CqbHLH144*) showed a significant positive correlation. We speculate that these two genes interact synergistically and coordinate their physiological functions under stress and in the reproductive stages. In conclusion, these results indicated that some functions of *CqbHLH* may be related to the network mechanisms between genes.

## 5 Conclusion

In this study, we systematically studied the *C. quinoa bHLH* gene family at the genome level and performed a series of analyses and verifications. 218 C*. quinoa bHLH* genes were analyzed, these *CqbHLH* genes were divided into 20 groups based on their protein motifs and gene structure, and 218 *CqbHLH* genes were irregularly distributed on 18 chromosomes and Chr00.Gene replication events may have produced some *CqbHLH* genes; fragment repeat contributes more to amplifying the *CqbHLH* gene family than tandem duplication. In terms of homology, a phenomenon was discovered that one *CqbHLH* gene and multiple *B. distachyon bHLH* genes are homologous. Among the six representative plants, the *CqbHLH* gene and *S. lycopersicum bHLH* genes have the most homologous pairs. qRT-PCR results showed that the 20 selected *CqbHLH* genes were affected by abiotic stress. *CqbHLH88* and *CqbHLH144* showed significant effects on abiotic stress resistance; *CqbHLH88* and *CqbHLH144* also have important effects on the reproductive development stages of *C. quinoa*. We speculate that *CqbHLH88* and *CqbHLH144* are greatly significant in the life cycle of *C. quinoa*. In summary, this study provides basic information on the biological function and bioinformatics of *C. quinoa*.

## Data availability statement

The entire Chenopodium quinoa genome sequence information was obtained from the Phytozome database (http://www.phytozome.net/). The datasets supporting the conclusions of this study are included in the article and its additional files. Further inquiries can be directed to the corresponding author.

## Author contributions

GX wrote the manuscript, and GX and YF analysed the data and designed the experiment. GX, YF, CZ, HY, LF, XC, YY, XY, WW, LK, and CL edit graphics and schedules. JC supervised the study, and JC and JR reviewed and revised the manuscript. All authors contributed to the article and approved the submitted version.

## Supplementary material

Supplementary Table 1 218 *CqbHLH* genes identified and protein sequences.

Supplementary Table 2 AtbHLH protein sequence for phylogeny.

Supplementary Table 3 The cis-acting element of the promoter sequence 2,000 bp upstream of 218 *bHLH* genes in *Chenopodium quinoa.*

Supplementary Table 4 The one pairs of tandem duplicates in *CqbHLH* genes.

Supplementary Table 5 The 95 pairs of segmental duplicates in *CqbHLH* genes.

Supplementary Table 6 One-to-one orthologous relationships between *Chenopodium quinoa* and 6 different plants.

Supplementary Table 7 bHLH protein sequences of six species.

Supplementary Table 8 218 Conserved Motifs of CqbHLH Protein and 7 species bHLH protein conserved motifs.

Supplementary Table 9 Primer sequences for qPCR.
